# The Biosynthetic Gene Cluster for Andrastin A in *Penicillium roqueforti*

**DOI:** 10.3389/fmicb.2017.00813

**Published:** 2017-05-05

**Authors:** Juan F. Rojas-Aedo, Carlos Gil-Durán, Abdiel Del-Cid, Natalia Valdés, Pamela Álamos, Inmaculada Vaca, Ramón O. García-Rico, Gloria Levicán, Mario Tello, Renato Chávez

**Affiliations:** ^1^Departamento de Biología, Facultad de Química y Biología, Universidad de Santiago de ChileSantiago, Chile; ^2^Departamento de Química, Facultad de Ciencias, Universidad de ChileSantiago, Chile; ^3^GIMBIO Group, Department of Microbiology, Faculty of Basic Sciences, Universidad de PamplonaPamplona, Colombia

**Keywords:** *Penicillium roqueforti*, fungal secondary metabolism, andrastin A, gene cluster, RNA-mediated gene silencing

## Abstract

*Penicillium roqueforti* is a filamentous fungus involved in the ripening of several kinds of blue cheeses. In addition, this fungus produces several secondary metabolites, including the meroterpenoid compound andrastin A, a promising antitumoral compound. However, to date the genomic cluster responsible for the biosynthesis of this compound in *P. roqueforti* has not been described. In this work, we have sequenced and annotated a genomic region of approximately 29.4 kbp (named the *adr* gene cluster) that is involved in the biosynthesis of andrastin A in *P. roqueforti*. This region contains ten genes, named *adrA, adrC, adrD, adrE, adrF, adrG, adrH, adrI, adrJ* and *adrK*. Interestingly, the *adrB* gene previously found in the *adr* cluster from *P. chrysogenum*, was found as a residual pseudogene in the *adr* cluster from *P. roqueforti*. RNA-mediated gene silencing of each of the ten genes resulted in significant reductions in andrastin A production, confirming that all of them are involved in the biosynthesis of this compound. Of particular interest was the *adrC* gene, encoding for a major facilitator superfamily transporter. According to our results, this gene is required for the production of andrastin A but does not have any role in its secretion to the extracellular medium. The identification of the *adr* cluster in *P. roqueforti* will be important to understand the molecular basis of the production of andrastin A, and for the obtainment of strains of *P. roqueforti* overproducing andrastin A that might be of interest for the cheese industry.

## Introduction

Andrastin A is a meroterpenoid compound produced by several fungi from the genus *Penicillium* (Nielsen et al., 2005; Sonjak et al., 2005; Visagie et al., 2014). This metabolite has interesting biological activities that make it a promising antitumoral compound. Andrastin A inhibits the farnesyltransferase activity of the oncogenic Ras proteins, and also promotes the intracellular accumulation of anticancer compounds in tumoral cells (Uchida et al., 1996; Rho et al., 1998).

Regarding the biosynthesis of andrastin A by fungi, early studies (Uchida et al., 1996) suggested that this compound is derived from a farnesyl pyrophosphate and, at that time, an unknown tetraketide (later known as 3,5-dimethylorsellinic acid, DMOA). More recently, a genomic cluster (the *adr* cluster) that is responsible for the andrastin A biosynthesis in *Penicillium chrysogenum*, was identified (Matsuda et al., 2013). The *adr* cluster from *P. chrysogenum* contains eleven genes named *adrA* (encoding a cytochrome P450 monooxygenase), *adrB* (encoding a protein with unknown function), *adrC* (encoding a putative major facilitator superfamily (MSF) transporter), *adrD* (encoding a polyketide synthase, PKS), *adrE* (encoding a ketoreductase), *adrF* (encoding a short chain dehydrogenase/reductase), *adrG* (encoding a prenyltransferase), *adrH* (encoding a FAD-dependent monooxygenase), *adrI* (encoding a terpene cyclase), *adrJ* (encoding an acetyl transferase) and *adrK* (encoding a methyl transferase) (Matsuda et al., 2013).

Based on the fact that *adrD, adrG, adrK*, and *adrH* have high similarity to their respective homologous genes of the austinol and terretonin biosynthetic gene clusters, Matsuda et al. (2013) hypothesized the four first putative steps of the andrastin A biosynthesis in *P. chrysogenum*. They proposed that the four enzymes encoded by *adrD, adrG, adrK* and *adrH* would act consecutively to produce epoxyfarnesyl-DMOA methyl ester from the primary metabolites acetyl CoA, malonyl CoA and *S*-adenosylmethionine. From this point, and by using the heterologous co-expression of *adrI, adrF, adrE, adrJ*, and *adrA* in a strain of *Aspergillus oryzae* that produces epoxyfarnesyl-DMOA methyl ester, they were able to experimentally reconstitute the rest of the pathway until the formation of andrastin A (Matsuda et al., 2013). Thus, currently five out of the eleven *adr* genes from *P. chrysogenum* (*adrI, adrF, adrE, adrJ*, and *adrA*) have been experimentally shown to be involved in andrastin A biosynthesis, whereas other four genes (*adrD, adrG, adrK*, and *adrH*) are probably involved in the biosynthesis of this compound, but this has not been experimentally demonstrated yet. Finally, the putative roles of the *adrB* and *adrC* genes in andrastin A biosynthesis pathway remain as unknown.

*Penicillium roqueforti* is a filamentous fungus widely used in the production of blue-veined cheeses, such as Roquefort, Stilton, and others (Fernández-Bodega et al., 2009). In addition, and like other filamentous fungi, *P. roqueforti* is an active producer of secondary metabolites. Thus, this species produces mycotoxins (such as roquefortine C and PR-toxin), and bioactive compounds such as mycophenolic acid and andrastin A (García-Estrada and Martín, 2016). In *P. roqueforti*, the gene clusters responsible for the biosynthesis of roquefortine C, PR-toxin and mycophenolic acid have been recently identified (Kosalková et al., 2015; Del-Cid et al., 2016; Hidalgo et al., 2016). However, to date the genes responsible for the biosynthesis of andrastin A in this fungus remain as unknown. Interestingly, *P. roqueforti* produces andrastin A during cheese ripening (Nielsen et al., 2005; Fernández-Bodega et al., 2009). Taking into account the potential positive effects of andrastin A on human health, it has been proposed that strains of *P. roqueforti* overproducing this compound might be of interest for the future production of “functionalized cheeses” with higher quantities of andrastin A (Albillos et al., 2011). For these purposes, the knowledge of the genes involved in the biosynthesis of andrastin A in *P. roqueforti* would be very useful.

In the present work, we have sequenced and annotated a genomic region containing the biosynthetic gene cluster for andrastin A in *P. roqueforti*. In addition, we performed the functional characterization of each gene of that cluster by using RNA interference.

## Materials and Methods

### Fungal Strains and Culture Media

*Penicillium roqueforti* strain CECT 2905 (ATCC 10110) was used in this work. This strain and all the transformants obtained in this work were kept on Potato dextrose agar (Merck, Germany), excepting when andrastin A production was required. In these cases, the strains were grown on YES agar [Bacto Yeast Extract (Difco, USA) 20 g/L, sucrose (Merck, Germany, biochemical grade) 150 g/L and Bacto Agar (Difco, USA) 20 g/L].

### Sequencing and Identification of the Andrastin A Gene Cluster of *P. roqueforti* CECT 2905

DNA from *P. roqueforti* CECT 2905 was obtained as described before (Gil-Durán et al., 2015). This DNA was used to perform the sequencing of the *P. roqueforti* CECT 2905 genome, by using Illumina technology. This genome was assembled in several contigs and currently is under annotation (unpublished data). We take advantage of this genome sequence to identify the *adr* cluster from *P. roqueforti* CECT 2905 as follows: the *adrD* gene from *P. chrysogenum* (7,930 nucleotides) encoding the putative PKS, was used to scan all the contigs from *P. roqueforti* CECT 2905 by BlastN. One contig containing a gene with very high similarity to *adrD* (coverage 99%, identity 84%, and *E*-value closer to zero) was obtained. Then, the vicinities of this gene were compared to the rest of the *adr* cluster from *P. chrysogenum* by BlastX, BlastP, and BlastN, revealing the presence of the rest of the *adr* genes, excepting *adrB* (see Results). All the genes were manually analyzed, delimited, and annotated using a combination of the Blast tools, multiple alignments by Clustal Omega, and the Translate Tool from Expasy web interface^1^.

The nucleotide sequence of the *adr* gene cluster from *P. roqueforti* CECT 2905 described in this work has been deposited in the GenBank database under accession number KY349137.

### Construction of RNA-Silencing Plasmids and Transformation of *P. roqueforti*

To silence the ten genes of the *adr* cluster of *P. roqueforti*, RNA-mediated gene silencing technology was used. The strategy was essentially the same described by Del-Cid et al. (2016). Briefly, a small sequence of each gene was amplified by PCR using suitable primers (Supplementary Table S1). Each amplicon was digested with *Nco*I and then, ligated into plasmid pJL43-RNAi (Ullán et al., 2008) previously digested with the same restriction enzyme, thus giving rise to ten RNAi-silencing constructs (pJL-RNAi-adrA to pJL-RNAi-adrK, Supplementary Table S1 and Figure S1). These constructs were then used to transform *P. roqueforti*, exactly as was described before (Gil-Durán et al., 2015).

### RT-qPCR Experiments

For RT-qPCR experiments, total RNA was purified as described previously (Gil-Durán et al., 2015) and quantified in a MultiSkan GO quantification system (Thermo Scientific, Germany). Two μg of RNA were used to synthesize cDNA using RevertAid Reverse Transcriptase (Thermo Scientific, Germany). For each RT-qPCR reaction (20 μl) the following conditions were set: 10 μl of KAPA SYBR Fast qRT-PCR Master Mix 2x (Kapa Biosystems, USA), 0.4 μl of each primer (at a concentration of 10 μM each), 0.4 μl de 50x ROX High/Low, 6.8 μl of water and 2 μl of the cDNA previously synthesized. RT-qPCR reactions were carried out in the StepOne Real-Time PCR System (Applied Biosystems, USA). Amplification conditions were 20 s at 95°C and 40 cycles of 3 s at 95°C and 30 s at 50°C. Three replicates were performed for each analysis and suitable negative controls were included. Relative gene expression values were determined by the comparative Ct (ΔΔCt) method using β-tubulin gene expression as a normalization control. The sequences of the primer sets used in RT-qPCR experiments are described in the Supplementary Table S2.

### Extraction of Andrastin A and HPLC Analysis

The extraction of andrastin A was done using the same method described by Del-Cid et al. (2016). Briefly, the fungal strains were grown on YES agar for 7 days at 28°C. The selected sample (mycelium or triturated agar) was extracted overnight with 50 mL of an ethyl acetate: dichloromethane: methanol (3:2:1) mixture containing formic acid (1%). After that, the mixtures were sonicated during 30 min and filtered through a 0.45 μm Millex-HV hydrophilic PVDF syringe filter (Merck Millipore). The filtrated was evaporated to dryness in a rotary evaporator, resuspended in 500 μl of methanol (HPLC grade) and submitted to HPLC analysis. The HPLC equipment used consists in a Waters 1525 HPLC system (Waters, Ireland) equipped with a Waters 1525 Binary HPLC pump, a Waters 2996 Photodiode Array (PDA) Detector and a 4.6 × 250 mm (5 μm) SunFire C18 column. HPLC runs were performed as was described by Del-Cid et al. (2016): samples (20 μL) were injected into the HPLC using water (solvent A) and acetonitrile (solvent B), both acidified with 0.02% trifluoroacetic acid. The elution gradient was as follows: 15% solvent B to 68% solvent B linear over 25 min, 68% solvent B to 100% solvent B linear over 2 min, isocratic for 5 min and 100% solvent B to 15% solvent B linear over 2 min. The flow used was 1.2 mL/min and the column was held at 35°C.

Under the HPLC conditions described above, andrastin A was identified by the “spiking” technique. Briefly, known amounts of pure andrastin A (Santa Cruz Biotechnology, Dallas, TX, USA) were added to the samples (spiked samples). As control, non-spiked samples were used. In all the spiked samples, a single peak (with retention time of 24.72 min) increased its size. The increase of the peak size was proportional to the amount of pure andrastin A added. No increase of the peak size was observed in non-spiked samples and no new chromatographic peaks or “shoulder peaks” were seen in the spiked samples. Moreover, the identity of the peak was confirmed by UV-Vis absorption spectrum (200–600 nm). Both the UV-Vis spectrum and the retention time of the peak were identical to those observed for the pure andrastin A.

Finally, the quantity of andrastin A of each sample was obtained from a calibration curve constructed with the pure compound as standard and the UV detector set at 254 nm. The quantity of andrastin A obtained was normalized to the dry weight of the fungal mycelia. For this purpose, the mycelium of each *P. roqueforti* strain was dried as described before (García-Rico et al., 2009).

## Results

### Identification and Bioinformatics Analysis of the Andrastin A Gene Cluster in *Penicillium roqueforti* CECT 2905

**FIGURE 1:**
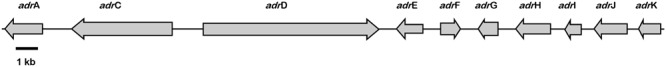
**Schematic organization of the *adr* gene cluster in *P. roqueforti* CECT 2905.** The arrows represent the genes and the direction of their transcription. The organization of the *adr* cluster in *P. roqueforti* FM164 (Cheeseman et al., 2014) is identical (data not shown).

To find the *adr* gene cluster in *P. roqueforti* CECT 2905, we sequenced and scanned its genome as was described in Material and Methods. As a result, a genomic region of approximately 29.4 kbp containing 10 genes was identified (**Figure 1**). This region has high similarity (84% overall identity) to the *adr* gene cluster from *P. chrysogenum* (Matsuda et al., 2013). Thus, according to the nomenclature of their orthologs in *P. chrysogenum*, the genes found in the *P. roqueforti adr* cluster were named *adrA, adrC, adrD, adrE, adrF, adrG, adrH, adrI, adrJ*, and *adrK* (**Figure 1**). At this point, it should be noted that recently, the genome of another *P. roqueforti* strain (named FM164) was sequenced and annotated (Cheeseman et al., 2014). We also analyzed this genome and as expected, we found the *adr* cluster. The *adr* clusters from both *P. roqueforti* strains have identical size, contain the same 10 genes with identical organization, and are almost identical at nucleotide level (only 75 nucleotide differences in 29,321 total nucleotides, representing 99% overall identity, 100% coverage and no gaps). In **Table 1**, the correspondence between each *adr* gene from strain CECT 2905 and the respective ORF from the genome of strain FM164 is described.

**Table 1:** Analysis of the deduced proteins encoded by the *adr* cluster of *P. roqueforti* CECT 2905.

Gene name in *P. roqueforti*							
**Strain CECT2905^a^**	**Strain FM164^b^**	**Size of the deducedprotein^c^ (aminoacids)**	**Putative function in andrastin A biosynthesis^d^**	**Identity (%) withorthologous proteins ofthe *P. chrysogenum adr* cluster**
*adrA*	Proq04g062820	508	Cytochrome P450 monooxygenase	94
*adrC*	Proq04g062830	1,452	MFS transporter	83
*adrD*	Proq04g062840	2,495	Polyketide synthase	83
*adrE*	Proq04g062850	336	Ketoreductase	89
*adrF*	Proq04g062860a	256	Short chain dehydrogenase	91
*adrG*	Proq04g062870	316	Prenyltransferase	87
*adrH*	Proq04g062880a	476	FAD-dependent monooxygenase	83
*adrI*	Proq04g062890	245	Terpene cyclase	94
*adrJ*	Proq04g062900	496	Acetyltransferase	85
*adrK*	Proq04g062910	278	Methyltransferase	94

*^a^The *adr* gene cluster from *P. roqueforti* CECT 2905 can be found at GenBank under accession number KY349137. ^b^Gene nomenclature according to the original annotation of the genome of strain FM164 (Cheeseman et al., 2014). In this genome, the *adr* gene cluster is contained in the genomic scaffold ProqFM164S01 (GenBank accession number HG792015). ^c^The sizes of the deduced proteins in strains CECT 2905 and FM164 are identical. ^d^Putative functions of the orthologous proteins of the *P. chrysogenum adr* cluster (Matsuda et al., 2013).*

Despite several efforts, we did not found any ORF with similarity to the *adrB* gene from *P. chrysogenum* in the *P. roqueforti* genome. Specifically, we used the *adrB* gene from *P. chrysogenum* and its deduced protein to exhaustively scan the whole genome of *P. roqueforti* CECT 2905 using several bioinformatics analysis (BlastN, BlastX, tBlastN) with no positive results. It should be noted that this gene was not found either in the genome of *P. roqueforti* strain FM164 (Cheeseman et al., 2014), suggesting that its absence is a common fact in *P. roqueforti* strains.

**FIGURE 2:**
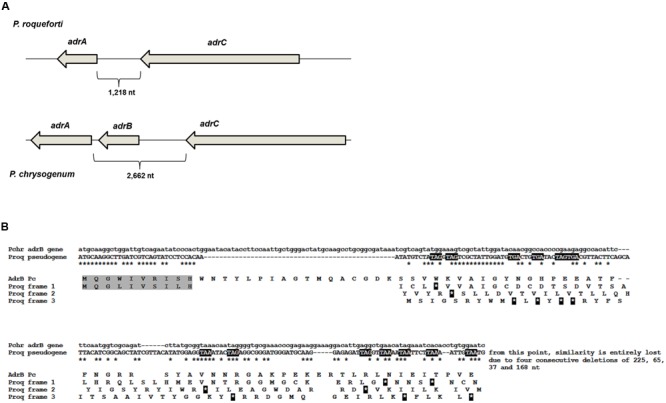
**Comparison between the zones comprising *adrA* to *adrC* in *P. roqueforti* and *P. chrysogenum*. (A)** Schematic comparison between these zones. The drawings are at scale. In *P. chrysogenum*, the *adrB* gene is included. Please note the difference in the size of these regions between *P. roqueforti* and *P. chrysogenum*. **(B)** Alignment of the nucleotide sequence (5′ end) of the *adrB* gene from *P. chrysogenum* (PchradrB gene) and the pseudogene found in *P. roqueforti* (Proq pseudogene). The first 30 nucleotides (and their 10 deduced aminoacids, highlighted in gray box) show high similarity. However, a deletion of 49 nucleotides in the pseudogene (plus other minor deletion/insertion events) results in several in-frame translations stop codons (highlighted in black boxes). As a consequence, after translation, protein fragments resulting show very low similarity to AdrB from *P. chrysogenum* in the three reading frames. The sequence of the pseudogene shown is the only part that shows some degree of similarity. After this sequence, and due to four consecutive deletions in the pseudogene, the similarity between these regions is entirely lost.

Recently, it has been suggested that some fungal gene clusters can suffer reorganization processes leading to the total or partial loss of some genes (Martín and Liras, 2016). In accordance with this suggestion, it can be hypothesized that the *adrB* gene originally present in *P. chrysogenum* was entirely or partially lost in the *adr* cluster from *P. roqueforti*. To address this hypothesis, we take the region comprising the intergenic zone between *adrA* and *adrC* in *P. roqueforti* and we compared it with the syntenic region from the *P. chrysogenum* genome. A first interesting observation was that the intergenic zone between *adrA* and *adrC* in *P. roqueforti* is 1,444 nucleotides shorter that the syntenic region from *P. chrysogenum* (1,218 bp vs. 2,662 bp; **Figure 2A**), supporting the possibility that the *adrB* gene was total or partially lost in *P. roqueforti*. Using these regions, we performed several alignments using nucleotide and protein sequences, and we found that the *adrB* gene in *P. roqueforti* was partially lost and is found as a residual pseudogene (**Figure 2B**). In this pseudogene, the first 10 aminoacids almost exactly match to the first 10 aminoacids of the AdrB protein from *P. chrysogenum* (**Figure 2B**). However, from this point, a deletion of 49 nucleotides (and other minor insertion/deletion events) changes the sequence and produces in-frame stop codons in all the frame shifts (**Figure 2B**). Finally, and due to additional four consecutive deletion events in the pseudogene, the similarity between these sequences is entirely lost.

Finally, regarding the deduced proteins encoded by the *adr* genes from *P. roqueforti*, **Table 1** summarizes the sizes and putative functions of these proteins. As can be observed, all the deduced proteins have high similarity to their respective orthologs from *P. chrysogenum* (between 83 and 94% identities), suggesting that in *P. roqueforti*, these proteins should perform the same function assigned in *P. chrysogenum*.

### RNA-Mediated Silencing of the Ten Genes of the *adr* Cluster from *Penicillium roqueforti* CECT 2905

To test the participation of each gene of the *adr* cluster from *P. roqueforti* CECT 2905 in andrastin A biosynthesis, we employed RNA-mediated gene-silencing technology. This technology has been widely used to demonstrate the functionality of several biosynthetic gene clusters in *P. roqueforti* (Kosalková et al., 2015; Del-Cid et al., 2016; Hidalgo et al., 2016). For this purpose, ten suitable plasmids were constructed and used to transform *P. roqueforti* CECT 2905 (see Material and Methods for details).

**FIGURE 3:**
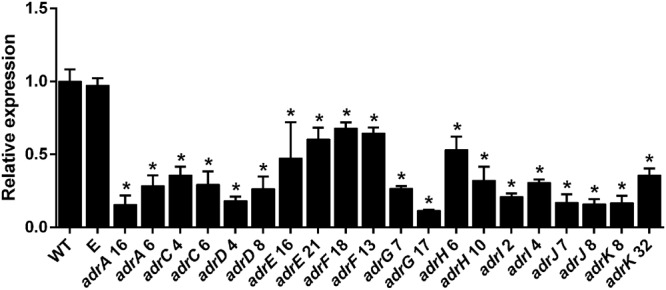
**qRT-PCR analysis of the expression of *adr* genes in the RNAi-silenced transformants of *P. roqueforti* CECT 2905.** Two transformants were selected for each gene. They were adrA6, adrA16, adrC4, adrC6, adrD4, adrD8, adrE16, adrE21, adrF13, adrF18, adrG7, adrG17, adrH6, adrH10, adrI2, adrI4, adrJ7, adrJ8, adrK 8 and adrK 32. As controls, *P. roqueforti* CECT 2905 (WT) and *P. roqueforti* CECT 2905 containing empty pJL43-RNAi vector (E) were used. Error bars represent the standard deviation of three replicates in three different experiments. The symbol ^∗^indicates that reductions in the expression of the genes in RNAi-silenced transformants were statistically significant (*P* < 0.05 using Student’s *t*-test) respect to the wild-type strain and *P. roqueforti* CECT 2905 containing empty pJL43-RNAi.

In each transformation event, around 40–50 phleomycin-resistant transformants were obtained. From each transformation event, fifteen transformants were chosen randomly and submitted to preliminary RT-PCR analysis (data not shown). Those two transformants showing the most significant decrease in the mRNA level were selected for further quantification of the down-regulation using RT-qPCR (**Figure 2**). The results indicate that depending on the gene, the transformants selected exhibited between 1.4- and 10- fold of decrease in mRNA levels compared with the wild-type strain of *P. roqueforti* (**Figure 3**), confirming the successful knock-down of all the genes of the *adr* cluster. Additionally, in all cases the presence of the full silencing cassette was confirmed (Supplementary Figure S1). The transformants selected were used for further analysis.

### Effect of *adrI, adrF, adrE, adrJ*, and *adrA* Silencing on Andrastin A Production in *Penicillium roqueforti* CECT 2905

**FIGURE 4:**
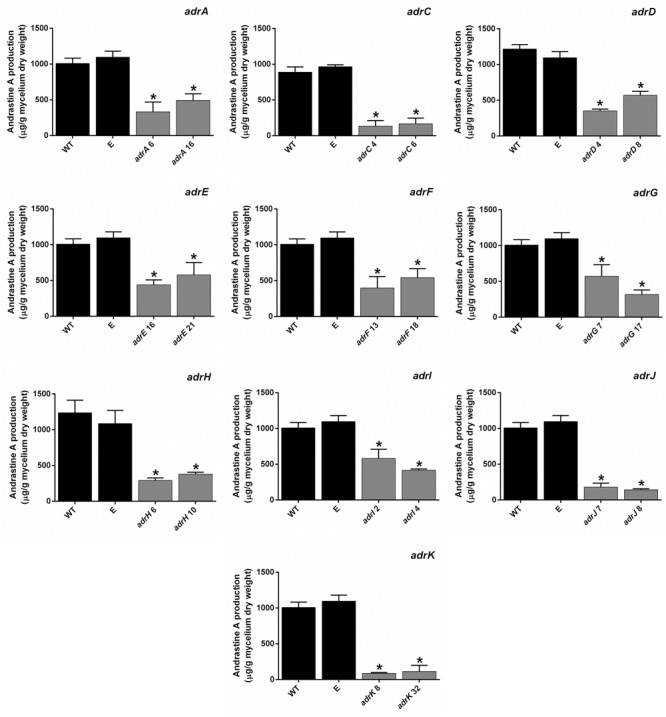
**Production of andrastin A by *P. roqueforti* strains.** For a best comparison, data for each gene is shown separately. RNAi-silenced transformants are the same described in **Figure 3**. Metabolites were extracted, quantified and normalized by the dry weight of the fungal mycelia as described in Section “Materials and Methods.” Error bars represent the standard deviation of three replicates in three independent experiments. The symbol ^∗^indicates that reductions in andrastin A production by RNAi-silenced transformants were statistically significant (*P* < 0.05 using Student’s *t*-test). As can be observed, production of andrastin A by *P. roqueforti* CECT 2905 containing empty pJL43-RNAi vector was statistically indistinguishable from the wild-type strain.

Previously, the participation of *adrI, adrF, adrE, adrJ* and *adrA* genes in the biosynthesis of andrastin A in the fungus *P. chrysogenum* was experimentally demonstrated (Matsuda et al., 2013). Therefore, as first approach, we tested if their orthologs in *P. roqueforti* are effectively involved in the andrastin A biosynthesis. For this purpose, RNA-mediated silenced transformants were obtained (see above) and they were used to evaluate the production of andrastin A by HPLC (**Figure 4**). As control, *P. roqueforti* CECT 2905 (wild-type strain) and a strain harboring empty plasmid pJL43-RNAi were used.

Silencing of the five genes aforementioned drastically reduced the production of andrastin A by *P. roqueforti* CECT 2905 (**Figure 4**). Specifically, transformants with attenuated levels of *adrI, adrF, adrE, adrJ* and *adrA* transcripts produced between 14 and 57.7% of the andrastin A produced by the wild-type strain, depending on the gene and the transformant analyzed (**Figure 4**). These results experimentally confirm the participation of these genes in the production of andrastin A by *P. roqueforti*.

### Effect of *adrD, adrG, adrK*, and *adrH* Silencing on Andrastin A Production in *Penicillium roqueforti* CECT 2905

It has been hypothesized that *adrD, adrG, adrK* and *adrH* encode for four enzymes that would act consecutively in the four first putative steps of the andrastin A biosynthesis (Matsuda et al., 2013). However, to the best of our knowledge, the role of these genes has not been experimentally tested yet. Therefore, it was of great interest to address the participation of these genes in the biosynthesis of andrastin A in *P. roqueforti*.

Silencing of the *adrD, adrG, adrK*, and *adrH* genes drastically reduced the production of andrastin A by *P. roqueforti* CECT 2905 (**Figure 4**). Specifically, transformants with attenuated levels of *adrD, adrG, adrK*, and *adrH* transcripts produced between 8.6 and 56.7 % of the andrastin A produced by the wild-type strain, depending on the gene and the transformant analyzed (**Figure 4**). These results provide the first experimental support for the participation of *adrD, adrG, adrK* and *adrH* in the *in vivo* production of andrastin A by fungi.

### The *adrC* Gene, Encoding for a MFS Transporter, Is Required for the Production of Andrastin A But Does Not Have a Significant Role in Its Secretion

The protein encoded by *adrC* has high similarity with MFS transporters proteins (**Table 1**). MSF proteins are ubiquitous membrane proteins that are responsible for the movement of a wide range of substrates across biological membranes (Quistgaard et al., 2016). In the case of genes encoding for MSF proteins located into fungal biosynthetic gene clusters, it has been suggested that these proteins could be involved in the secretion of the secondary metabolites produced (Martín et al., 2005; Martín and Liras, 2016). Therefore, we tested if *adrC* could influence andrastin A production and/or secretion. Interestingly, the silencing of *adrC* drastically reduced the production of andrastin A by *P. roqueforti* CECT 2905 (**Figure 3**). The transformants with attenuated levels of *adrC* transcripts produced between 15.2 and 18.7 % of the andrastin A produced by the wild-type strain (**Figure 4**), suggesting that *adrC* is necessary for the production of andrastin A.

**FIGURE 5:**
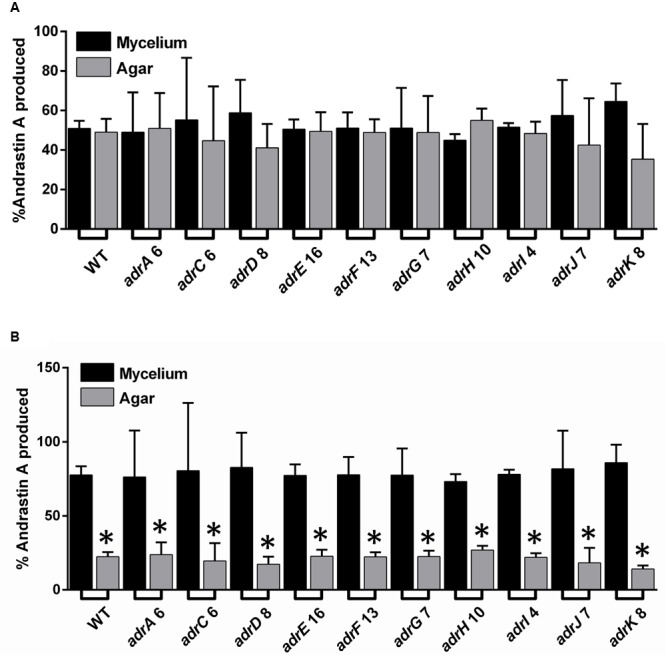
**Comparison of the levels of andrastin A found in mycelium and agar in *P. roqueforti* strains. (A)** Comparison of the quantity (μg) of andrastin A found in mycelium and agar. For simplicity, only one transformant is shown for each gene. Each strain was grown on solid YES medium for 7 days, and andrastin A was extracted separately from both the mycelium and agar as described in Section “Materials and Methods.” In each case, the quantity (μg) of andrastin A found in mycelium and agar is shown as percentage (%) of the total quantity of andrastin A produced by the strain. Error bars represent the standard deviation of three replicates in three independent experiments. As can be observed, in all cases no significant differences were found between the percentage of andrastin A in the mycelium and the agar (Student’s *t*-test, *P* < 0.05). **(B)** Comparison of the concentrations (μg/mL) of andrastin A in mycelium and agar. Andrastin A was quantified as above, and in each case, the volume of the mycelium or agar was used to calculate concentration. Results are shown as percentages. Error bars represent the standard deviation of three replicates in three different experiments. The symbol ^∗^indicates that the differences in andrastin A concentration between mycelium and the agar were statistically significant (*P* < 0.05 using Student’s *t*-test).

Regarding a putative role of *adrC* in andrastin A secretion, we compared the quantity (μg) of the compound found in mycelium and agar medium (**Figure 5A**). The result, expressed as the percentage of the total andrastin A produced by the strain, indicates that the quantity of andrastin A found in agar and mycelium is similar in the wild type strain and the transformants with attenuated levels of *adrC* transcripts. The same result was obtained when all the rest of strains with *adr* genes attenuated were analyzed (**Figure 5A**). In addition, in order to assess a putative effect of the volume of the sample used (agar or mycelium) we also compared the concentrations (μg/mL) of andrastin A in these samples. Our results indicate that the concentrations of andrastin A in the cells of the transformants with attenuated levels of *adrC* transcripts are around three times higher than those found in agar (**Figure 5B**). However, this effect was not specific to *adrC* and was observed in all the strains, including the wild-type strain (**Figure 5B**). Taken together, these results suggest that *adrC* (or any other gene from the *adr* cluster) has no specific role in andrastin A secretion.

## Discussion

*Penicillium roqueforti* strain CECT 2905 produces several secondary metabolites and currently, the gene clusters responsible for the biosynthesis of three of them, namely roquefortine C, PR-toxin and mycophenolic acid, have been identified (Kosalková et al., 2015; Del-Cid et al., 2016; Hidalgo et al., 2016). Here we describe the gene cluster responsible for the biosynthesis of another secondary metabolite from this fungus, andrastin A, a promising antitumoral compound. In *P. roqueforti* CECT 2905, the *adr* gene cluster comprises a genomic region of approximately 29.4 kbp and contains ten genes. More interesting, the silencing of all of them resulted in significant reductions in andrastin A production, confirming their involvement in the biosynthesis of this compound.

As was stated before (see Introduction), Matsuda et al. (2013) experimentally reconstituted the last steps in the formation of andrastin A from the intermediate epoxyfarnesyl-DMOA methyl ester, by using the heterologous co-expression of *adrI, adrF, adrE, adrJ* and *adrA* genes from *P. chrysogenum* in an *A. oryzae* strain that produces the mentioned intermediate. In agreement with these results, here we show the participation of their orthologous genes in the biosynthesis of andrastin A by *P. roqueforti*. In *P. chrysogenum, adrI, adrF, adrE, adrJ*, and *adrA* encode for a terpene cyclase, a short chain dehydrogenase/reductase, a ketoreductase, an acetyl transferase and a cytochrome P450 monooxygenase, respectively. All these proteins from *P. chrysogenum* have very high similarity to their orthologs from *P. roqueforti* (**Table 1**), strongly suggesting that they may catalyze the same reactions in the biosynthetic pathway of andrastin A in *P. roqueforti*.

Regarding the four enzymes encoded by *adrD, adrG, adrK* and *adrH*, it has been hypothesized that they would act consecutively in the four first steps of the andrastin A biosynthesis (Matsuda et al., 2013). However, to the best of our knowledge, the participation of these genes in the biosynthesis of andrastin A has not been experimentally tested thus far in any fungus. Our results provide the first experimental support for the participation of these genes in the *in vivo* production of andrastin A by fungi.

Compared with *P. roqueforti*, the *adr* cluster from *P. chrysogenum* contains one additional gene named *adrB*, which was found as a residual pseudogene in the *P. roqueforti adr* cluster (**Figure 2**). Recently, Martín and Liras (2016) have suggested that during their evolutionary formation, some fungal gene clusters can suffer drastic reorganization processes. As a product of this reorganization, some genes could be entirely lost, whereas other genes could be partially lost and found as residual pseudogenes. In accordance with this proposal, our results suggest that the *adrB* gene originally present in *P. chrysogenum* was partially lost in the *adr* cluster from *P. roqueforti*. The loss of genes in a *P. roqueforti* gene cluster, compared with the respective *P. chrysogenum* cluster, has been observed before in the roquefortine C/ meleagrin gene cluster. In *P. chrysogenum*, this cluster contains seven genes, whereas in *P. roqueforti* it is shorter and contains four genes (Kosalková et al., 2015). In this case, the evolutionary reorganization of the cluster produced that two genes present in *P. chrysogenum* were entirely lost in *P. roqueforti*, whereas a third gene was partially lost and now is found as a residual pseudogene (Kosalková et al., 2015; Martín and Liras, 2016). These data and our observation about the pseudogenization of *adrB* in the *adr* cluster suggest that the evolutionary reorganization in clusters from *P. chrysogenum* and *P. roqueforti* may be a common and extended process.

Taking into account the pseudogenization of *adrB* in *P. roqueforti*, an interesting question arises: is *adrB* a functional gene in *P. chrysogenum*? In the *P. chrysogenum* genome (GenBank accession number AM920437), the *adrB* gene corresponds to ORF Pc22g22830 of 1,162 bp, encoding a protein of 232 aminoacids annotated as “hypothetical protein.” In accordance with that, Matsuda et al. (2013) did not assign any function to the *adrB* gene in the andrastin A biosynthetic pathway in *P. chrysogenum*. We performed our own BlastP search using the deduced AdrB protein, and we did not found similarity with any protein in the whole GenBank database (data not shown). In a first view, these data suggest that *adrB* may represent an ORF defined by *in silico* annotation, but it may not be a functional gene from *P. chrysogenum*. However, some evidences call into question this explanation. Specifically, two transcriptomic assays by microarray performed on *P. chrysogenum* have found expression of mRNAs from ORF Pc22g22830 (van den Berg et al., 2008; Becker et al., 2016), indicating that *adrB* is actively transcribed in *P. chrysogenum*. Thus, taking into account that at least at transcriptional level, *adrB* seems to be a functional gene in *P. chrysogenum*, in the future it would be interesting to test if *adrB* has any role in andrastin A biosynthesis in this fungus.

Our results suggest that the *adrC* gene encoding for a putative MSF transporter is necessary for the production of andrastin A by *P. roqueforti*. In literature, there are other cases where genes encoding MSF proteins are necessaries for the production of secondary metabolites by fungi. For example, in *Cercospora kikuchii* and *Fusarium fujikuroi*, the disruption of genes encoding MSF transporters leads to drastic reductions in the production of cercosporin and bikaverin, respectively (Callahan et al., 1999; Wiemann et al., 2009). Currently, it remains unclear how the disruption of genes encoding for MSF proteins can reduce the levels of production of fungal secondary metabolites. Callahan et al. (1999) suggested that the lack of MSF protein leads to the accumulation of the secondary metabolite above a critical threshold, producing the inhibition of biosynthetic enzymes or the down-regulation of the transcription of the biosynthetic genes. Another possibility is that these MSF proteins be intracellular transporters involved in the traffic of some intermediates of the biosynthesis (Martín and Liras, 2016). The absence of these transporters could be interfering in the normal intracellular transport of these intermediates, hence decreasing the production of the final secondary metabolite.

Interestingly, despite the *adrC* gene is required for the production of andrastin A, it does not have any role in the secretion of the compound to the extracellular medium. Although there are several cases where MSF transporters are clearly linked to the secretion of a given fungal secondary metabolite (see reviews of Martín et al., 2005; Martín and Liras, 2016), there are several other cases where the MSF transporters does not have role in the secretion of secondary metabolites. For example, when the *aflT* gene (encoding for a MFS transporter within the aflatoxin gene cluster in *A. parasiticus*) was disrupted, the deleted mutants secreted aflatoxins at similar levels to the wild type strain (Chang et al., 2004). Similarly, when the *mfsA* gene from *A. carbonarius* (encoding a MFS transporter in the ochratoxin A gene cluster) was deleted, ochratoxin A was found at similar levels both in mycelia and extracellular medium in all the strains analyzed (Crespo-Sempere et al., 2014). The same was observed in the case of the *roqT* gene from the roquefortine C/meleagrin cluster in *P. chrysogenum*: mutants disrupted in this MSF-encoding gene were still able to secrete roquefortine C, indicating that the protein encoded by *roqT* is not involved in the secretion of this compound (Ali et al., 2013).

According to our results, none of the *adr* genes from *P. roqueforti* is in charge of the secretion of andrastin A to the extracellular medium, so the secretion of this compound must to proceed by other mechanism. The simplest mechanism is passive diffusion across the plasmatic membrane. However, other active mechanisms cannot be ruled out. Chanda et al. (2009) showed that once synthesized, aflatoxin is exported to the extracellular medium by an exocytosis process leading by specialized vesicles, named aflatoxisomes. On the other hand, in *P. roqueforti* it has been suggested that the secretion of roquefortine C could be performed by additional redundant MSF transporters (the so-named “surrogate transporters,” Kosalková et al., 2015). In the future, it will be very interesting to investigate whether any of these mechanisms may be responsible of andrastin A secretion in *P. roqueforti*.

## Author Contributions

IV, RG-R, GL, MT, and RC conceived and designed the experiments, contributed reagents/materials, analyzed the data and supervised work. JR-A, CG-D, AD-C, PA and IV carried out the experiments and analyzed the data. NV, IV, and MT performed genome sequencing, assembly, annotation, and genome analysis. JR-A, CG-D, AD-C, PA, and RC performed bioinformatics analysis. PA, IV, GL, and RC drafted the manuscript. All authors have read and approved the manuscript.

## Conflict of Interest Statement

The authors declare that the research was conducted in the absence of any commercial or financial relationships that could be construed as a potential conflict of interest.

## Footnotes

1 http://web.expasy.org/translate/
