# Supplementary material for: The Biosynthetic Gene Cluster for Andrastin A in Penicillium roqueforti
Source: Front Microbiol. 2017 May 5;8:813. doi: 10.3389/fmicb.2017.00813 (PMC5418334; doi:10.3389/fmicb.2017.00813)
Supplement: Supplementary file 1 [file Data_Sheet_1.pdf]

Table S1. Name of each primer obtained, size of the insert ligated in each plasmid, and sequences of the primers used to amplify each insert.

Plasmid	Size of the insert (bp)	Sequence of primers (5`--3`) used in amplification of inserts*
pJL-RNAi-adrA	382	Fw: AGACTCCCATGGTTTGCCGTAATCCCGACT
		Rv: AGACTCCCATGGCGTCCACAAAGATCAAGG
pJL-RNAi-adrC	412	Fw: AGACTCCCATGGACCAGCTAGAACTTACTC
		Rv: AGACTCCCATGGTCGATTAGAGCAGTGGAT
pJL-RNAi-adrD	324	Fw: AGACTCCCATGGCACCAGATACCGAAGCAG
		Rv: AGACTCCCATGGTTGTGCATCGCAGACGCT
pJL-RNAi-adrE	287	Fw: AGACTCCCATGGCTTGGTTTGTGGATGTGG
		Rv: AGACTCCCATGGACCGCTCTCTAGGCTGAC
pJL-RNAi-adrF	401	Fw: AGACTCCCATGGATCACCGGTAGCGCCTCT
		Rv: AGACTCCCATGGACCACTCGTAGCTGCCT
pJL-RNAi-adrG	329	Fw: AGACTCCCATGGTTGCCTTATAATCACATT
		Rv: AGACTCCCATGGCCATCGCGAGGAATCCGG
pJL-RNAi-adrH	325	Fw: AGACTCCCATGGGTAGTTCACGCAGATGCC
		Rv: AGACTCCCATGGTCCACTGCTGCTGAGGCC
pJL-RNAi-adrI	374	Fw: AGACTCCCATGGATCATAGAGATGCCTTGG
		Rv: AGACTCCCATGGCCCAACCTCTCGCGCAAA
pJL-RNAi-adrJ	394	Fw: AGACTCCCATGGGCCCCAACTATCTATACA
		Rv: AGACTCCCATGGCTCTTCTTTCCCGCGTGA
pJL-RNAi-adrK	371	Fw: AGACTCCCATGGTCCTTGAGCTTGGCCTTG
		Rv: AGACTCCCATGGGTTGTCGACTTTTAGCTT

* *Nco*I sites are highlighted in red.

Table S2. Primers used in qRT-PCR experiments

Target gene	Name of primer	Sequence 5´---3´	Amplicon size (pb)
adrA	adrA-qPCR fw	CGACTGGCTACGTCTCACTG	106
	adrA-qPCR rv	TAAAAACCTAGCCGCGATTG	
adrC	adrC-qPCR fw	TGTGTCAGATCCTCGTTGAA	85
	adrC-qPCR rv	CTGCCGTAAACCGAAAAGTA	
adrD	adrD-qPCR fw	GGCTCGGACGACTATACTGA	84
	adrD-qPCR rv	AGTACAGAACGCCTGGAGTG	
adrE	adrE-qPCR fw	CTCGTATTCCAGCTGCTCCT	94
	adrE-qPCR rv	CCAAAAGTCTCACGAAGCAA	
adrF	adrF-qPCR fw	CCATGAGACGACACCAAGGC	112
	adrF-qPCR rv	ATCAACGCTGGAATTCTGGT	
adrG	adrG-qPCR fw	GAGTCCATCGAGCTCCTGTC	98
	adrG-qPCR rv	ACGAAAAATGACAGGCCAAG	
adrH	adrH-qPCR fw	GACACCCAATATCGGACAAG	93
	adrH-qPCR rv	AAGGCATCTGCGTGAACTAC	
adrI	adrI-qPCR fw	ACGTCGCGAAAAGACAAGAT	97
	adrI-qPCR rv	TCGCGGTTGGGTAGATAAAG	
adrJ	adrJ-qPCR fw	TGCGTGGACTGCTCTACTTC	82
	adrJ-qPCR rv	GAGTTCTGTGAGCGGGTCTA	
adrK	adrK-qPCR fw	TGACCCAAAGAGCCTCTACA	88
	adrK-qPCR rv	CGCTGGAATCTTGCTGTATT	

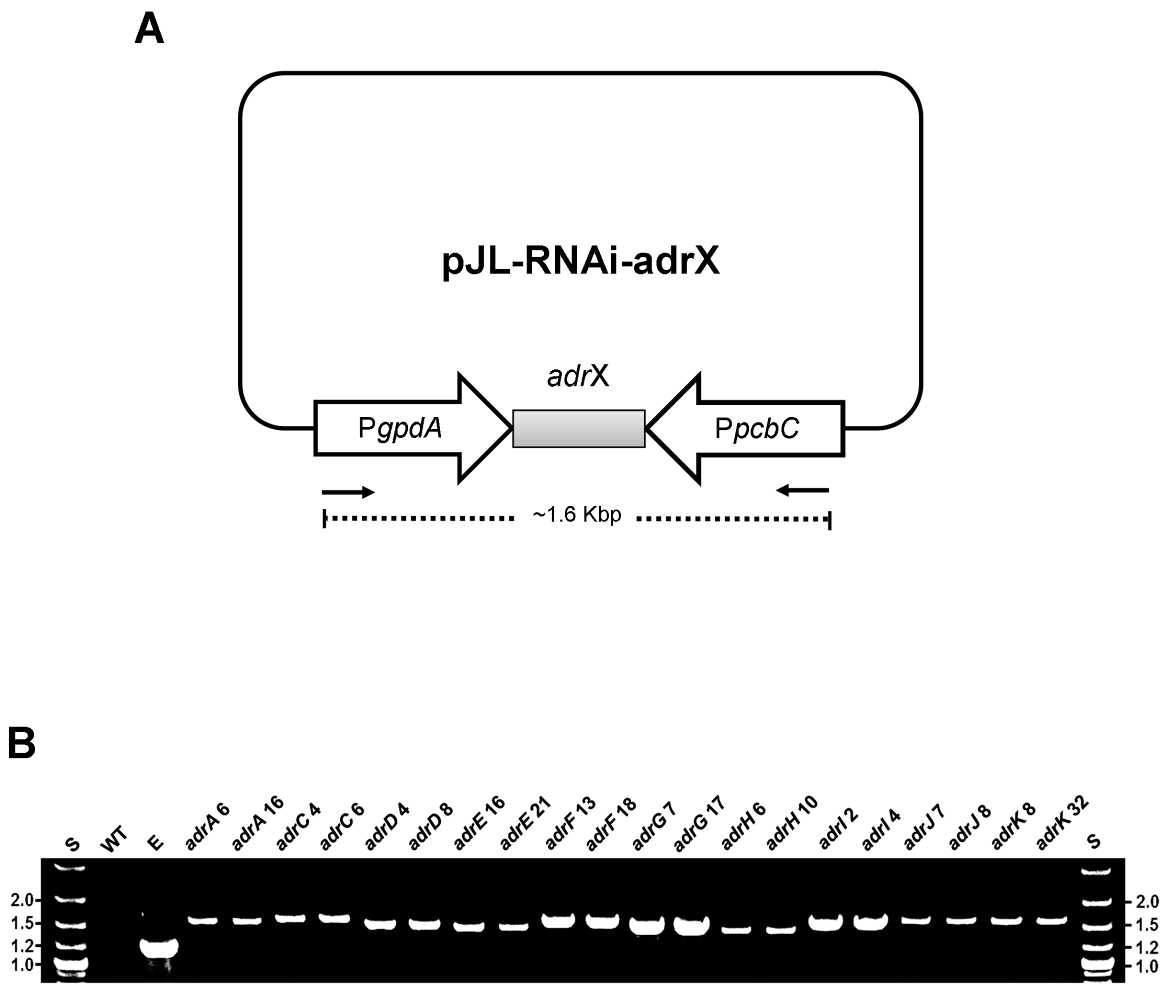

Figure S1. A) Example of the plasmid constructed for each gene from the *adr* cluster. P*gpdA*: promoter from the *gpd* gene from *A. nidulans*. P*pcbC*: promoter from the *pcbC* gene from *P. chrysogenum*. Both promoters are oriented in opposite directions, generating double-stranded RNA molecules (dsRNAs) from the DNA fragment inserted, and thus triggering the fungal RNA-silencing machinery. The grey box between the promoters represents a fragment from a given *adr* gene (*adr*X, where X identifies the specific gene; see Supplementary Table S1). The small black arrows represent primers ConfRNAiFW (5`- GCATGCCATTAACCTAGG-3`) and ConfRNAiRV (5`-ACGGTGGCTGAAGATTC-3`). These primers were used to confirm the integration of the full silencing cassette (see panel B). The expected size of the amplicon containing the full silencing cassette (around 1.6 Kbp) is indicated. B) PCR assay demonstrating integration of the full silencing cassette in each transformant obtained. Amplicons obtained were subjected to electrophoresis in agarose gels. Lane WT: wild-type strain *P. roqueforti* CECT 2905; lane E: *P. roqueforti* CECT 2905 containing empty pJL43-RNAi vector; lane S: Standard GeneRuler 1 kb DNA Ladder (Fermentas). Relevant sizes expressed in kb are shown at left. Please note that as expected, length of amplicon obtained from *P. roqueforti* CECT 2905 containing empty vector is shorter (expected size 1.2 Kbp).
